# Supplementary material for: IL-36 signaling in keratinocytes controls early IL-23 production in psoriasis-like dermatitis
Source: Life Sci Alliance. 2020 Apr 28;3(6):e202000688. doi: 10.26508/lsa.202000688 (PMC7190273; doi:10.26508/lsa.202000688)
Supplement: Supplementary file 2 [file LSA-2020-00688_TableS2.docx]

**Table S2. Function of validated genes from RNASeq.** Genes in bold are up-regulated. Genes underlined are down-regulated

| **IL-36-dependent, KC36-dependent genes (d3)** | | |
| --- | --- | --- |
| **Gene** | **KEGG pathways found enriched** | **Some other known functions** |
| ***Ly6g*** | None | Lymphocyte antigen 6 complex, locus G. Specific marker of neutrophils. Might be involved in their migration |
| ***Csf3*** | Cytokine-cytokine receptor interaction, JAK/STAT signaling pathway, IL-17 signaling pathway, Hematopoietic cell lineage, PI3K/Akt signaling pathway, Malaria | Colony stimulating factor 3 (granulocyte) (G-CSF). Proliferation, differentiation and trafficking of neutrophils. Induced by IL-36 and IL-17A in keratinocytes |
| ***Il22*** | Cytokine-cytokine receptor interaction, JAK/STAT signaling pathway, Inflammatory bowel disease (IBD), Th17 cell differentiation | Produced by T cells or innate lymphoid cells stimulated by IL-23. Inhibits keratinocyte terminal differentiation. Induces psoriasis pathogenic signature and exacerbates disease in synergy with other factors. Critical cytokine for Aldara^®^-induced psoriasis |
| ***Cxcl3*** | IL-17 signaling pathway, NOD-like receptor signaling pathway, TNF signaling pathway, Salmonella infection, Chemokine signaling pathway, Legionellosis, Kaposi sarcoma’s associated herpes virus infection | Chemokine (C-X-C motif) ligand 3. Powerful neutrophil chemoattractant. Involved in wound healing. Induced by IL-17 in keratinocytes |
| ***U90926*** | None | cDNA sequence U90926. Long non coding RNA. Negative regulator of antigen presentation in human bone marrow-derived macrophages (BMM) and dendritic cells (BMDC) |
| ***Cxcl2*** | Cytokine-cytokine receptor interaction, IL-17 signaling pathway, NOD-like receptor signaling pathway, TNF signaling pathway, Salmonella infection, Chemokine signaling pathway, Legionellosis, NF-kappa B signaling pathway, Kaposi sarcoma’s associated herpesvirus infection | Chemokine (C-X-C motif) ligand 2. Upregulated in psoriatic skin. Powerful neutrophil chemoattractant. Induced by IL-36 and IL-17A in keratinocytes |
| ***Slc26a4*** | Thyroid hormone synthesis | Solute carrier family 26, member 4. Expressed in inner ear. Downregulated expression causes hearing loss |
| ***Il12b*** | Cytokine-cytokine receptor interaction, Jak/STAT signaling pathway, Inflammatory bowel disease (IBD), Herpes simplex infection, Influenza A, Toll-like receptor signaling pathway, Legionellosis, Pertussis, Tuberculosis, Measles, Chagas disease, Amoebiasis, Th1 and Th2 cell differentiation, African trypanosomiasis, Allograft rejection, Type I diabetes mellitus, RIG-1-like receptor signaling pathway, Toxoplasmosis, Proteoglycans in cancer, Leishmaniasis | Interleukin 12b. Common subunit of IL-12 and IL-23 |
| ***Il17f*** | Cytokine-cytokine receptor interaction, Inflammatory bowel disease (IBD), IL-17 signaling pathway, Th17 cell differentiation | Interleukin 17F. Often co-expressed with IL-17A, forms heterodimers with it and signals through the same receptor. High expression in psoriatic skin. IL-17F-deficient mice are more resistant than IL-17A-deficient mice to Aldara^®^-induced psoriasis-like skin inflammation. Induces IL-8 and IL-6 production in human keratinocytes |
| ***Acod1*** | None | Aconitate decarboxylase 1. Mediates itaconate production in macrophages, contributing to their anti-microbial activity. Itaconate inhibits IL-17-mediated IκBζ expression in keratinocytes and ameliorates Aldara^®^-induced psoriasis-like skin inflammation |
| ***Cd300lf*** | None | CD300 molecule like family member F. Expressed in myeloid cells and B cells. LPS-induced expression in BMDC and neutrophils. Inhibits IL-6 production in vitro and DC-mediated specific T cell responses in vitro and in vivo. Induces apoptosis of myeloid cells. In humans, the locus containing CD300LF gene has been associated with psoriasis |
| ***Il23a*** | Cytokine-cytokine receptor interaction, Jak/STAT signaling pathway, Inflammatory bowel disease (IBD), Rheumatoid arthritis, Th17 cell differentiation, Pertussis, Tuberculosis | Interleukin 23, alpha subunit p19. Expressed by activated immune cells, keratinocytes, Langerhans cells, dermal dendritic cells and macrophages. Enhanced expression in lesion psoriatic skin. Keratinocyte-produced IL-23 is sufficient to cause chronic skin inflammation with IL-17 signature. Induces IL-17 production |
| ***Slfn4*** | None | Schlafen 4. Expressed during macrophage activation by TLR agonists in BMM, but down-regulated during differentiation. Induced by Type I interferons. Overexpression induces reduction of monocytes and inflammatory macrophages numbers in peritoneal cavity. Expressed by myeloid-derived suppressor cells inhibiting T cell responses in murine gastric metaplasia |
| ***Spdef*** | None | SAM pointed domain containing ets transcription factor. Expressed by gut, lung and stomach epithelial cells. Involved in mucus-producing goblet cells differentiation and Th2 inflammation. Involved in intestinal crypt cells proliferation. Tumor suppressor. Indirect elements suggesting expression in keratinocytes. Low expression in skin |
| ***Mmp3*** | IL-17 signaling pathway, Rheumatoid arthritis, TNF signaling pathway, Transcriptional misregulation in cancer | Matrix metallopeptidase 3. Involved in keratinocyte migration. Expressed in basal keratinocytes and macrophages of psoriatic skin. High levels in patients sera. Involved in cell migration and tissue remodeling. Induced by IL-17 and IL-22 |
| ***Lilr4b*** | None | Leukocyte immunoglobulin-like receptor, subfamily B, member 4B. Highly expressed by tolerogenic APCs. Induced in myeloid DCs by vitamin D. Indirectly required for induction of Treg cells. Increased expression in healing psoriatic lesions after treatment with calcipotriol |
| *Rasl10a* | None | RAS-like, family 10, member A. Overexpression in cell lines promotes cell cycle arrest and caspase-independent cell death. Tumor suppressor in neural cell lines |
| *Plin1* | PPAR signaling pathway, Apelin signaling pathway, Regulation of lipolysis in adipocytes | Perilipin 1. Required for optimal lipid homeostasis. Expression correlates with differentiation of hamster sebocytes. Induced expression by dupilumab treatment of atopic dermatitis patients. Downmodulated in epidermis of patients with psoriasis and eczema |
| *Car3* | Nitrogen metabolism | Carbonic anhydrase 3. Antioxidative agent. Induced in differentiating human epidermal keratinocytes. Present in atopic dermatitis skin |
| **IL-36-dependent, KC36-independent genes (d3)** | | |
| **Gene** | **KEGG pathways** | **Some other known functions** |
| ***Cxcr1*** | Cytokine-cytokine receptor interaction, Chemokine signaling pathway, Endocytosis, Phospholipase D signaling pathway | Chemokine (C-X-C motif) receptor 1. High expression on neutrophils. Also expressed by monocytes or T cells. Mediates neutrophil infiltration into inflamed tissues |
| ***Gm4758 (=Cstdc3)*** | None | Cystatin domain containing 3. Unknown function |
| ***Il17a*** | Cytokine-cytokine receptor interaction, Inflammatory bowel disease (IBD), IL-17 signaling pathway, Rheumatoid arthritis, Th17 cell differentiation | Interleukin 17a. Expressed by T cells, neutrophils and ILCs, notably upon IL-23 stimulation.  Induces aberrant proliferation and altered differentiation of keratinocytes. Induces expression of neutrophil chemo-attractants and of genes from psoriasis signature in keratinocytes, including IL-36. Acts also on endothelial cells, fibroblasts and chondrocytes |
| ***Trem1*** | None | Triggering receptor expressed on myeloid cells 1. Constitutively expressed on monocytes and neutrophils. Induced by LPS stimulation in HaCaT human keratinocyte cell line. Induces production of TNFα and IL-8. Expressed in circulation and lesional skin of psoriatic patients, and decreased expression after successful treatment |
| ***Fgf23*** | PI3K/Akt signaling pathway, Regulation of actin cytoskeleton, MAPK signaling pathway, Breast cancer, Pathways in cancer, Ras signaling pathway, Melanoma, Rap1 signaling pathway | Fibroblast growth factor 23. Found elevated in serum of psoriatic patients. Principal regulator of phosphate homeostasis. Expressed mainly by osteocytes and osteoblasts but also found in thymus and lymph nodes. Function in psoriasis unknown |
| ***Il6*** | Cytokine-cytokine receptor interaction, JAK/STAT signaling pathway, Inflammatory bowel disease (IBD), IL-17 signaling pathway, NOD-like receptor signaling pathway, Rheumatoid arthritis, Herpes simplex infection, Th17 cell differentiation, TNF signaling pathway, Influenza A, Salmonella infection, Toll-like receptor signaling pathway, Legionellosis, Pertussis, Hematopoietic cell lineage, PI3K/Akt signaling pathway, Tuberculosis, Cytosolic DNA sensing pathway, Measles, Chagas diseae (American tryponomiasis), Transcriptional misregulation in cancer, Malaria, Amoebiasis, Kaposi sarcoma’s associated herpesvirus infection, Hypertrophic cardiomyopathy (HCM), African trypanosomiasis, Insulin resistance, Graft versus host disease, EGFR tyrosine kinase inhibitor resistance, HIF-1 signaling pathway, HTLV-1 infection, Antifolate resistance, Hepatitis B, Non-alcoholic fatty liver disease, Prion diseases, Intestinal immune network for IgA production, Pathways in cancer, AGE-RAGE signaling pathways in diabetic complications, FoxO signaling pathway | Interleukin 6. Produced by keratinocytes, fibroblasts, endothelial cells, DC, macrophages and Th17 cells. Strongly associated with psoriasis. Induces keratinocyte growth, activation and production of pro-inflammatory cytokines, differentiation of Th17 cells, production pf pro-inflammatory cytokines and chemokines by macrophages and DC, and neutrophil differentiation. IL-23-induced psoriasis is prevented in IL-6-deficient mice. Hypothesized to have a regulatory effect on other pro-inflammatory cytokines with worst impact on psoriasis pathology |
| ***2010005H15Rik (=Csta2)*** | None | Cystatin A family member 2. Unknown function. Family member of cystatin A, which is expressed in keratohyaline granules of the stratum granulosum and in the cornified envelope of the epidermis. Cystatin A is also overexpressed in psoriasis skin |
| ***Reg3g*** | None | Regenerating islet-derived 3 gamma. Overexpressed in keratinocytes during psoriasis, wound healing and Aldara^®^-induced skin inflammation in an IL-17A-dependent manner. Induces keratinocyte proliferation and inhibits their differentiation |
| ***Trem3*** | None | Triggering receptor expressed on myeloid cells 3. Expressed in macrophages and T cells. Activating receptor. Upregulated by LPS, TNFα and IL-1β in hepatic macrophages and endothelial cells. No known correlation with psoriasis, despite close homology with *Trem1* (see above) |
| ***Mcpt8*** | None | Mast cell protease 8. Marker of mast cells and basophils |
| ***Sell*** | Cell adhesion molecules | Selectin, lymphocyte. CD62L. Type-I transmembrane glycoprotein and cell adhesion molecule expressed in most leukocytes. Mandatory for trafficking of naïve T cells to lymph nodes. Aldara^®^-induced psoriasis-like skin inflammation was significantly reduced in *Sell*-deficient mice |
| ***Clec4e*** | Tuberculosis | C-type lectin domain family 4, member e. Expressed by myeloid cells, notably macrophages. Recognizes glycolipid PAMPs from pathogens. Inhibits macrophage and neutrophil apoptosis in *A. fumigatus* keratitis. Involved in neutrophils’ NET formation during pneumoseptic infection with *Klebsiella pneumoniae*. Key role in neutrophil migration and resistance during polymicrobial sepsis. Could be involved in Malassezia-dependent inflammation in psoriatic patients’ skin |
| ***Gml*** | None | Glycosylphosphatidylinositol anchored molecule like. Belongs to Ly6 gene family. Hematopoietic cell-specific transcript. No known association with psoriasis |
| ***Cd177*** | None | CD177 antigen. Belongs to Ly6 gene family. Expressed exclusively in some neutrophil subsets and involved in their migration by binding to CD31. Expressed notably by an IL-17-producing subset, enriched in blood of allergic asthmatic patients. Potential TREM-1 ligand |
| ***Mmp1b*** | IL-17 signaling pathway, Rheumatoid arthritis, PPAR signaling pathway, Bladder cancer, Pathways in cancer | Matrix metallopeptidase 1b (interstitial collagenase). Cleaves fibrillary collagens type I-III. Increases mobility of dermal fibroblasts and epidermal keratinocytes. Involved in psoriasis pathogenesis. High levels in patient’s skin and serum. Expressed in basal and suprabasal keratinocytes, dermal fibroblasts and infiltrated immune cells (monocytes and macrophages). Modification of the extracellular matrix. Involved in cell migration and tissue remodeling. Induced by IL-17 and IL-22 |
| ***Spatc1*** | None | Spermatogenesis and centriole associated 1. Sperm centrosome protein. Shares a domain with an isoform of TNIP-1, a suppressor of TLR response, which mutations are associated with psoriasis. TNIP1 notably inhibits the IL-23/IL-17/IL-22 axis |
| *Ypel4* | None | Yippee like 4. Localised in centrosome and nucleolus during interphase. Might regulate MAPK signaling pathway to induce adrenal cell proliferation. Upregulated in lesional skin of psoriasis patients compared to healthy donors |
| *Colec11* | Phagosome | Collectin sub-family member 11. Innate immunity serum protein. Binds to *Escherichia coli*, *Candida albicans* or Influenza A virus. Expressed in many tissues including skin. Upregulated in skin T effector cells from mice colonized with *Staphylococcus aureus*, which also showed enhanced IL-17 expression |
| *Nnat* | None | Neuronatin. Neuronal developmental and differentiation molecule. Expressed in normal human epidermis and cultured keratinocytes. Overexpressed in psoriasis hyperplasic epidermis. Induced in differentiating keratinocytes. Involved in involucrin expression |
| *B3galt2* | Metabolic pathways, Glycosphingolipid biosynthesis | UDP-Gal:betaGlcNAc beta 1,3-galactosyltransferase, polypeptide 2. Glycosyltransferase. Expressed in brain trigeminal ganglion. Involved in expression of TLR4 and NK-κB, and production of TNFα and IL-6 in trigeminal ganglion neurons. Downregulated in human memory CD4^+^ T cells stimulated with anti-CD3/CD28 in presence of an inverse agonist of RORγt |
| *Cys1* | None | Cystin 1. Expressed in renal cilia. A mutation in the gene is responsible for development of autosomal recessive polycystic kidney disease in BALB/c-cpk/cpk mice. Found upregulated in skin samples from yellow nail disease patients. No correlation with psoriasis |
| *Itga11* | PI3K/Akt signaling pathway, Arrhythmogenic right ventricular cardiac myopathy (ARVC), ECM-receptor interaction, Hypertrophic cardiomyopathy (HCM), Regulation of actin cytoskeleton, Focal adhesion, Dilated cardiomyopathy | Integrin alpha 11. Collagen-binding integrin. Involved in cell migration and matrix organization. Expressed in human endothelial cells, chondrocytes and dermal fibroblasts. Marker of mesenchymal cells. Type I-IFN-responsive gene. Importantly induced during wound healing, involved in wound contraction |
| *Sema3d* | Axon guidance | Sema domain, immunoglobulin domain (Ig), short basic domain, secreted, (semaphorin) 3D. Secreted glycoprotein expressed in the developing parathyroid gland and thymus in mice. Inhibits parathyroid cell proliferation. Upregulated by TNF-α during the process of RANKL-induced osteoclast differentiation. Inhibits cell motility and migration of human endothelial cells, A regulatory intronic SNP of SEMA3D has been identified as a strong enhancer in epidermal keratinocytes. Decreased expression in skin from Aldara^®^-induced psoriasis-like skin inflammation. Downmodulated in psoriasis lesional compared to non-involved skin |
| *Cgn* | Tight junction | Cingulin. Tight junction actin-binding protein. Expressed in human skin endothelial cells, epidermis and keratinocytes. Overexpression induces tight junction formation and strengthens barrier function |
| *Aox1* | JAK/STAT signaling pathway, Nicotinate and nicotinamide metabolism, Metabolic pathways, Vitamin B6 metabolism, Retinol metabolism, Tyrosine metabolism, Tryptophan metabolism, Valine, leucine and isoleucine degradation, Drug metabolism – cytochrome P450 | Aldehyde oxidase 1. Drug-metabolizing enzyme, oxidizing xenobiotics. Upregulated in fibroblasts under oxidative stress. Treatment of DCs with sulforaphane, an anti-inflammatory compound, induces *Aox1* expression and represses *Il23a*, *Il12a* and *Il12b* expression |
| *Dbp* | None | D site albumin promoter binding protein. Robust marker for clock output. Influences circadian behavior. Involved in detoxification and cell metabolism. Mediates oxidative stress-induced apoptosis in fibroblasts. Important upregulation during early anagen within the secondary hair germ, which goes with keratinocyte proliferation |
| *Tmem233* | None | Transmembrane protein 233. From Dispanin family. Differentially expressed in psoriatic skin from patients compared to healthy donors. Unknown function |
| *Itga8* | PI3K/Akt signaling pathway, Cell adhesion molecules, Arrhythmogenic right ventricular cardiac myopathy (ARVC), ECM receptor interaction, Hypertrophic cardiomyopathy (HCM), Regulation of actin cytoskeleton, Focal adhesion, Dilated cardiomyopathy | Integrin alpha 8. Dimerizes with integrin β1. Involved in epithelialization of kidney mesenchymal cells. Involved in hair cell differentiation and inner ear stereocilia maturation. Differentially expressed in lesional and non-lesional skin from psoriatic patients compared to healthy donors. Upregulation in monocyte aggregates from psoriasis patients |
| *Flg2* | None | Filaggrin family member 2. Expressed in keratinizing epithelia. Increased expression in differentiating keratinocytes, Might contribute to epidermal barrier function. RNA interference-mediated FLG2 knockdown in a 3-dimensional reconstructed human epidermis resulted in thinner epidermis, parakeratosis, impaired stratum corneum, and abnormal vesicles inside corneocytes. Induced by GATA-3 overexpression in keratinocytes, GATA-3 which is downregulated in psoriasis epidermis. Decreased expression in tissue samples from psoriasis patients |
| *Pnpla3* | Metabolic pathways, Glycerolipid metabolism | Patatin-like phospholipase domain containing 3. Promotes transfer of essential fatty acids from triglycerides to phospholipids in hepatic lipid droplets. Marker of melanocytes. A mutation in *Pnpla3* confers the risk of developing non-alcoholic fatty liver disease, and psoriasis patients might suffer from a more severe form |
| *Hsd3b6* | Metabolic pathways, Steroid hormone biosynthesis, Ovarian steroidogenesis, Aldosterone synthesis and secretion | Hydroxy-delta-5-steroid dehydrogenase, 3 beta- and steroid delta-isomerase 6. Hypertension risk factor. Expressed in aldosterone-producing cells under the control of the circadian clock. Induced in skin of mouse undergoing wound healing. In healthy skin, expression is restricted to sebaceous glands |
| *Krt77* | None | Keratin 77. Expressed in human skin. Importantly expressed in early development of mouse epidermis, in correlation with the initiation of expression of other epidermal differentiation markers like K1 and loricrin, and with the initiation of barrier formation. Upregulated in glucocorticoid receptor-deficient mice embryos, which present defective epidermal differentiation. Expression importantly reduced in a model of reconstruction of human lesional psoriatic skin compared to reconstruction of non-lesional skin. Reduced expression in psoriatic lesions compared to uninvolved skin. Increased expression with mice deficient for glucocorticoid and mineralocorticoid receptors, which are more sensitive to Aldara^®^-induced psoriasis-like skin inflammation |
| *Lce1m* | None | Late cornified envelope 1M. Part of a genomic region called epidermal differentiation complex, of crucial importance for keratinocyte differentiation and stratum corneum properties. Expressed in the later stages of epithelial differentiation and incorporated into the cornified envelope. Increased expression in loricrin-deficient epidermis, including keratinocytes, as a compensatory mechanism. Downmodulated during keratinocyte differentiation |
| **IL-36-dependent, KC36-dependent genes (d7)** | | |
| **Gene** | **KEGG pathways** | **Other known functions** |
| ***Serpinb3d*** | Amoebiasis | Serine (or cysteine) peptidase inhibitor, clade B (ovalbumin), member 3D. Serine or cystine proteinase inhibitor. Expressed in epithelial tissues including keratinocytes, blood and immune cells. Induced by IL-22 and IL-17. Protects keratinocytes against UV-induced apoptosis. Transgenic expression increased epidermal proliferation. Autoantibodies recognizing Serpinb found in psoriatic patients sera. Increased expression found in psoriasis skin. Expression levels were correlated to the presence of parakeratotic cells in the stratum corneum. Patients with elevated expression were more susceptible to epidermal barrier disruption.  *NB: Information is given for Serpinb3, without distinction of isoforms. Nothing known on specific Serpinb3d function* |
| ***Serpinb3c*** | Amoebiasis | Serine (or cysteine) peptidase inhibitor, clade B, member 3C. Non-functional form of Serpinb3. See *Serpinb3d* for more details on Serpinb3 functions |
| ***A530032D15Rik*** | None | RIKEN cDNA A530032D15Rik gene. Ortholog of SP140 with unknown function. SP140 regulates cytokine production, inflammatory response and cell-cell adhesion |
| ***Hist1h4b (=H4c2)*** | Systemic lupus erythematosus, Alcoholism, Viral carcinogenesis | H4 clustered histone 2. Co-expressed and functionally coupled with Hinfp, an inducer of cell proliferation during embryogenesis |
| ***Fpr1*** | Staphylococcus aureus infection, Rap1 signaling pathway, Neuroactive ligand receptor interaction | Formyl peptide receptor 1. Expressed in neutrophils, macrophages and DCs. Expression induced by LPS in macrophages and neutrophils. Involved in myeloid cell migration and increased phagocytosis. In a mouse skin wound healing model, Fpr1/2 senses first chemotaxis signals, allowing rapid neutrophil infiltration. Critical for normal skin wound healing |
| ***Olfr56*** | Olfactory transduction | Olfactory receptor 56. Expressed in skin precursor cells |
| ***Ms4a8a*** | None | Membrane-spanning 4-domains, subfamily A, member 8A. Involved in calcium signaling, modulation of intracellular signaling and differentiation processes of haematopoietic and epithelial cells. Expressed in respiratory myeloid cells. Induced by TLR2/4/7 in association with M2 macrophage differentiation |
| ***Ly6i*** | None | Lymphocyte antigen 6 complex, locus I. Co-expressed with Ly6C in granulocytes and macrophages. Expressed in Ly6C^hi^ monocytes, immature B cells and in Ly6C^hi^ peripheral T cells |
| ***Gm6377*** | None | Predicted gene 6377. Unknown function |
| ***Sirpb1b*** | Osteoclast differentiation | Signal-regulatory protein beta 1B. Expressed in differentiating monocytes, macrophages, neutrophils and CD8^-^ DCs but not CD8^+^ DCs or plasmacytoid pre-DCs. Involved in cell to cell signaling and interaction, cellular function and maintenance, and hematological system development and function. Involved in neutrophils transepithelial migration |
| ***Xlr4b*** | None | X-linked lymphocyte-regulated 4B. Expressed in the brain. Th2 signature gene in mice |
| ***A530064D06Rik*** | None | RIKEN cDNA A530064D06 gene. Codes for PDC-TREM. Expressed on the pDC cell surface. Also expressed in BMDC, macrophages and splenic DC subsets. Preferentially expressed after TLR stimulation. Involved in IFNα production |
| ***Klrg1*** | None | Killer cell lectin-like receptor subfamily G, member 1. Immune checkpoint receptor expressed predominantly in late-differentiated effector and effector memory CD8^+^ T cells and blood NK cells. Identifies antigen-experienced T cells that are impaired in their proliferative capacity but are capable of performing effector functions. Recognizes E-cadherin and N-cadherin, expressed respectively on epithelial (including keratinocytes) and mesenchymal cells. Increased expression on NK cells, in periphery and spleen, in Aldara^®^-induced psoriasis |
| ***Pdcd1*** | Cell adhesion molecules (CAMs), T cell receptor signaling pathway, | Programmed cell death 1. Codes for PD-1, negative regulator of immune system expressed on T cells. Decreased frequencies of CD4^+^PD-1^+^ and CD8^+^PD-1^+^ T cells in psoriasis patients compared to control groups. Expressed on IL-17A^+^ TCRγδ^+^ T cells in psoriasis skin and Aldara^®^-treated mice. PD-L1-Fc injection alleviated psoriatic inflammation in Aldara^®^-treated mice. Psoriasis patients showed increased frequency and activation of circulating and lesional skin infiltrating Tfh cells, characterized by high expression of PD-1 |
| ***Spic1*** | None | Spindle And Centriole Associated Protein 1. Expression on myeloid cells is involved in regulation of Th17 cells in the context of dextran sodium sulfate-induced colitis, notably by inhibiting the expression of TLR-inducible genes. Expressed in B cells, where it downregulates NF-κB expression |
| *Fam57b* | None | Family with sequence similarity 57, member B. Expressed in chondrocytes. Produces lactosylceramide upon binding of dihydroxyvitamin D3. High expression in the skin. Might play a role in keratinocyte differentiation or wound repair |
| *Oxtr* | Neuroactive ligand-receptor interaction, Calcium signaling pathway, Oxytocin signaling pathway, cAMP signaling pathway | Oxytocin receptor. Receptor of the neuropeptide hormone oxytocin. Expressed in primary human dermal fibroblasts and keratinocytes. Inhibits proliferation of dermal fibroblasts and keratinocytes. Inhibits production of IL-6, CCL5 and CXCL10 in keratinocytes |
| *Alox12e* | Metabolic pathways, Serotonergic synapse, Arachidonic acid metabolism, Antifolate resistance, Inflammatory mediator regulation of TRP channels | Arachidonic lipoxygenase, epidermal. Lipid metabolism-related gene. Expressed in mouse epidermis |
| *Foxo6* | FoxO signaling pathway | Forkhead box O6. Transcription factor. Induces hepatic gluconeogenesis and increases fasting glycemia. Involved in macrophage infiltration into liver and adipose tissues by inducing CCR2 expression. Expressed in skin |
| *Chrm4* | Regulation of actin cytoskeleton, Neuroactive ligand-receptor interaction, Cholinergic synapse | Cholinergic receptor, muscarinic 4. Inhibitory receptor for acetylcholine. Expressed in peripheral blood lymphocytes, notably in B and T cells. Inhibits the production of pro-inflammatory cytokines. Expressed in keratinocytes. Involved in keratinocyte proliferation, adhesion and differentiation. Facilitates keratinocyte migration and wound re-epithelialization. Regulates the concentration of intracellular free Ca2^+^ in melanocytes |
| *Olig1* | None | Oligodendrocyte transcription factor 1. Smad cofactor involved in TGF-β-induced cell motility and wound healing assays |
| *Kazald1* | None | Kazal-type serine peptidase inhibitor domain 1. Upregulated in early phase of bone regeneration, Expressed in primary murine osteoblastic cells. Down-regulated in *NFKBIZ* (IL-36-controlled gene)-deficient primary keratinocytes after 24h of IL-36α stimulation |
| *Ffar4* | None | Free fatty acid receptor 4. Suppresses anti-inflammatory responses. Significantly improves tissue regeneration with thickened epithelium and accelerated wound repair.. Upon GPR120 activation, wound healing was significantly accelerated, with reduced production of IL-1β, and increased IL-6, TGF-β and involucrin.  Activation of GPR120 does not impact Aldara^®^-induced psoriasis |
| *Fmo2* | Drug metabolism – cytochrome P450 | Flavin containing monooxygenase 2. Expressed in minor human fibroblast populations, which are enriched in psoriasis lesional skin.  Expressed in the human skin, exclusively in the dermins, and high expression in a 3D-reconstructed model of human skin |
| *Lce1m* | None | Late cornified envelope 1M. Part of a genomic region called epidermal differentiation complex, of crucial importance for keratinocyte differentiation. Expressed in the later stages of epithelial differentiation and incorporated into the cornified envelope. Downmodulated during keratinocyte differentiation |
| *Crlf1* | None | Cytokine receptor-like factor 1. Member of the IL-6 family. B cell-stimulating factor. Up-regulated by TNF-α, IL-6 and IFN-γ in primary fibroblasts. Binds to IL-6Rα. Up-regulated by IL-36α treatment in *Nfkbiz* (downstream mediator of IL-36)-deficient mice compared to WT mice |
| *Msx2* | HTLV-1 infection | Msh homeobox 2. Expressed by keratinocytes. Induced by TNF-α. Overexpression induces thickening of the epidermis with hyperproliferation and hyperkeratosis. Regulates expression of hair keratins during hair follicle differentiation. Expressed in fetal skin then down-regulated, and re-expressed during wound healing. Msx2-deficient mice display faster wound closure with accelerated re-epithelialization plus earlier appearance of keratin markers. Msx2-deficient keratinocytes showed increased cell migration. Found differentially regulated in psoriatic skin of patients compared to healthy skin |
| *Cyp2j12* | Metabolic pathways, Serotonergic synapse, Arachidonic acid metabolism, Linoleic acid metabolism, Ovarian steroidogenesis, Inflammatory mediator regulation of TRP channels | Cytochrome P450, family 2, subfamily j, polypeptide 12. Catalyzes xenobiotics and endogenous compounds |
| *Krt2* | None | Keratin 2. Expressed in the ear. Expression downregulated under hyperplasia. Essential for the epidermal integrity of plantar skin. Deletion of K2 caused acanthosis and hyperkeratosis of the ear and the tail epidermis, corneocyte fragility, and local inflammation in the ear skin. Downregulated expression in psoriasis and hypertrophic scars where keratinocytes are known to undergo activation |
| *Bmp2* | Cytokine-cytokine receptor interaction, Pathways in cancer, Hippo signaling pathway, Basal cell carcinoma, TGF-beta signaling pathway | Bone morphogenic protein 2. Involved in fibroblast and adipocyte differentiation. Expressed in proliferative basal and differentiated suprabasal keratinocytes. Inhibits keratinocyte proliferation. Involved in wound healing. Controls cell growth arrest/terminal differentiation in normal primary human epidermal keratinocytes |
| *Hsd3b6* | Metabolic pathways, Steroid hormone biosynthesis, Ovarian steroidogenesis, Aldosterone synthesis and secretion | Hydroxy-delta-5-steroid dehydrogenase, 3 beta- and steroid delta-isomerase 6. Hypertension risk factor. Induced in skin of mouse undergoing wound healing |
| *Panx3* | None | Pannexin 3. Member of the pannexin gap junction protein family from tooth germs. Promotes chondrocyte differentiation. Modulated during keratinocyte differentiation. Overexpression reduced rat keratinocyte cell proliferation. Deficient mice exhibited a significant delay in wound healing with insufficient re-epithelialization, decreased inflammatory reaction, and reduced collagen remodeling. Involved in epithelial-mesenchymal transition during skin wound healing |
| *Nxph3* | None | Neurexophilin 3. Highly localized in cortical and cerebellar regions and functionally important for sensorimotor gating and motor coordination.  In damaged chicken ears, inhibition of γ-secretase (Notch pathway) enhances number of proliferative hair cells and expression of Nxph3 |
| *Mgll* | Metabolic pathways, Glycerolipid metabolism, Regulation of lipolysis in adipocytes, Retrograde endocannabinoid signaling | Monoglyceride lipase. Main enzyme implicated in the degradation of the most abundant endocannabinoid in the brain, 2-arachidonoylglycerol. Expressed in human CD16^+^ monocytes and upregulated compared to CD16^-^ monocytes. Marker of cell activation.  Deficiency results in lipid overload in tumor-associated macrophages and induces macrophage activation, which suppresses the function of tumor-associated CD8^+^ T cells |
| *Hs3st6* | None | Heparan sulfate (glucosamine) 3-O-sulfotransferase 6. Belongs to the keratinocyte differentiation gene network. Among top 15 of downregulated genes in psoriatic skin |
| *Ptpn21* | None | Protein tyrosine phosphatase, non-receptor type 21. Src-associated protein tyrosine phosphatase. Localizes along actin filaments and at adhesion plaques. Promotes the cytoskeleton events that induce cell adhesion and migration. Negatively regulates ICAM-1 expression in human keratinocytes by dephosphorylating IκKβ and blocking NF-κB activation. Transiently expressed in keratinocytes by TNFα stimulation. Key skin-differentiating functions |
| *Gm5127* | None | Predicted gene 5127. Highest expression in skin. Downregulated in flaky tail atopic dermatitis mouse model |
| *Tnnt3* | None | Troponin T3, skeletal, fast. Fast skeletal muscle isoform located in the muscle nucleus. Leads to muscle apoptosis. Downmodulated in skin of a mouse model of atopic dermatitis. Downregulated by estradiol treatment in epidermal keratinocytes |
| **IL-36-dependent, KC36-independent genes (d7)** | | |
| **Gene** | **KEGG pathways** | **Other known functions** |
| ***Ear1*** | None | Eosinophil-associated, ribonuclease A family, member 1. Elevated levels in psoriasis patients, in line with increased number of eosinophils in skin lesions. Induces production of IL-5, MMP-9 and CCL5, but not IL-17 or IL-31, by stimulated keratinocytes. Cytotoxic to keratinocytes, by inducing ROS formation and apoptosis. Induces significant keratinocyte detachment from provisional matrix |
| ***Il22*** | Cytokine-cytokine receptor interaction, Inflammatory bowel disease (IBD), Jak/STAT signaling pathway, Th17 cell differentiation | Interleukin 22. Produced by T cells, innate lymphoid cells or LTi-like cells stimulated by IL-23. Inhibits keratinocyte terminal differentiation. Induces psoriasis pathogenic signature and exacerbates disease in synergy with other factors. Involved in Aldara^®^-induced psoriasis |
| ***Stfa2l1*** | None | Stefin A2 like 1. Expression induced in Langerhans cells afer 5 days of Aldara^®^ treatment. Predicted partner of Evpl, Ppl and caspase-14, all three involved in epidermal differentiation |
| ***Gm4758 (=Cstdc3)*** | None | Cystatin domain containing 3. Unknown function |
| ***Gm5483 (=Cstdc4)*** | None | Cystatin domain containing 4. Upregulated in fetal skin from Rheb-deficient mice, which present profound skin barrier defects and impaired epidermal differentiation. Upregulated in Th22 cells compared to Th17 cells. |
| ***Il17a*** | Cytokine-cytokine receptor interaction, Rheumatoid arthritis, Inflammatory bowel disease (IBD), IL-17 signaling pathway, Th17 cell differentiation | Interleukin 17a. Expressed by T cells and ILCs, notably upon IL-23 stimulation.  Induces aberrant proliferation and altered differentiation of keratinocytes. Induces expression of neutrophil chemoattractants and of psoriasis signature genes in keratinocytes, including IL-36. Stimulates endothelial cells, fibroblasts and chondrocytes |
| ***Spdef*** | None | SAM pointed domain containing ets transcription factor. Expressed by gut, lung and stomach epithelial cells. Involved in mucus-producing goblet cells differentiation and Th2 inflammation. Involved in intestinal crypt cells proliferation. Indirect elements suggesting expression in keratinocytes. Low expression in normal skin |
| ***Il12b*** | Cytokine-cytokine receptor interaction, Inflammatory bowel disease (IBD), Amoebiasis, Legionellosis, Tuberculosis, Herpes simplex infection, Jak/STAT signaling pathway, Influenza A, Leishmaniasis, Toll-like receptor signaling pathway, Allograft rejection, Toxoplasmosis, Measles, Type 1 diabetes mellitus, Chagas disease (American trypanosomiasis), Pertussis, African trypanosomiasis, RIG-I.like receptor signaling pathway, Th1 and Th2 cell differentiation, Proteoglycans in cancer | Interleukin 12b. Common subunit of IL-12 and IL-23 |
| ***Saa1*** | None | Serum amyloid A 1. Highly expressed in psoriatic lesions and in skin from Aldara^®^-treated mice. Neutralization attenuates severity of psoriasis *in vivo*. Induces keratinocyte proliferation and expression of pro-inflammatory molecules (including pre and mature IL-1β, caspase 1, NLRP3 CCL20 and SAA1) while inhibiting keratinocyte differentiation *in vitro*. Induced by IL-17A, IL-1α and TNF-α in keratinocytes. Induces MMP-1 production in human dermal fibroblasts but not in keratinocytes. Induces production of IL-1β, IL-8. IL-10, IL-12 and IL-23 by granulocytes, lymphocytes and monocytes. Induces the release of mature IL-1β from neutrophils, mast cells and macrophages |
| ***Cxcl5*** | Cytokine-cytokine receptor interaction, Chemokine signaling pathway, Rheumatoid arthritis, IL-17 signaling pathway, Pertussis | Chemokine (C-X-C motif) ligand 5. Neutrophil chemoattractant. Induced by IL-36 and IL-17A in keratinocytes. Upregulated in peripheral blood cells from psoriatic arthritis and psoriasis patients. Involved in the increase severity of Aldara^®^-induced psoriasis in mice fed with high-fat diet. Induced by TNF-α in cutaneous mesenchymal stem cells derived from psoriasis patients. Upregulated in skin mesenchymal stem cells from psoriasis patients |
| ***Cxcl3*** | Chemokine signaling pathway, NOD-like receptor signaling pathway, Legionellosis, IL-17 signaling pathway, TNF signaling pathway, Kaposi’s sarcoma-associated herpesvirus infection, Salmonella infection | Chemokine (C-X-C motif) ligand 3. Powerful neutrophil chemoattractant. Involved in wound healing. Induced by IL-17 in keratinocytes |
| ***Slc26a4*** | Thyroid hormone synthesis | Solute carrier family 26, member 4. Expressed in inner ear. Downregulated expression causes hearing loss |
| ***Il17f*** | Cytokine-cytokine receptor interaction, Inflammatory bowel disease (IBD), IL-17 signaling pathway, Th17 cell differentiation | Interleukin 17F. Often co-expressed with IL-17A, forms heterodimers with it and signals through the same receptor. High expression in psoriatic skin. IL-17F-deficient mice are more resistant than IL-17A-deficient mice to Aldara^®^-induced psoriasis-like skin inflammation. Induces IL-8 and IL-6 production in human keratinocytes |
| ***Tmprss11b*** | None | Transmembrane protease, serine 11B. Increased expression in psoriatic epidermis. Induces cell growth and IL-8 production. Expressed by normal human keratinocytes |
| ***Cxcl2*** | Cytokine-cytokine receptor interaction, Chemokine signaling pathway, NOD-like receptor signaling, Legionellosis, NF-kappaB signaling pathway, IL-17 signaling pathway, TNF signaling pathway, Kaposi’s sarcoma-associated herpesvirus infection, Salmonella infection | Chemokine (C-X-C motif) ligand 2. Upregulated in psoriatic skin. Powerful neutrophil chemoattractant. Induced by IL-36 and IL-17A in keratinocytes |
| ***Adgrf1*** | None | Adhesion G protein-coupled receptor F1. Synaptamide receptor, involved in neurogenesis. Upregulated expression in psoriatic skin. High expression in keratinocytes |
| ***Il17c*** | Cytokine-cytokine receptor interaction, IL-17 signaling pathway | Interleukin 17c. Mainly expressed by epithelial cells in response to TNF or TLRs. Overexpressed in psoriatic skin. Induces production of pro-inflammatory cytokines, chemokines and anti-microbial peptides by skin and gut epithelial cells. Intradermal injection leads to epidermis thickening. IL-17C-deficient mice are more resistant to Aldara^®^-induced psoriasis. Regulated by IL-36γ in paradoxical psoriasis from IBD patients treated with anti-TNF. Blockade inhibits cutaneous inflammation in an IL-23-induced psoriatic-like skin inflammation model. Induced by TNFα in human keratinocytes. Overexpression in keratinocytes promotes psoriasiform skin inflammation |
| ***Nts*** | None | Neurotensin. Dopamine neuromodulator involved in schizophrenia. Induced by LPS in fetal-skin dendritic cells. Downregulates the expression of IL-6, TNFα, IL-10 and VEGF while upregulating EGF. Increased serum levels in psoriasis patients. Might play a role in psoriasis by activation of mast cells. Induces strong expression of miR-210 in colonic epithelial cells, which was shown to have a proinflammatory role by promoting inflammatory cytokine expression in CD4^+^ T cells from psoriasis patients |
| *Tmem233* | None | Transmembrane protein 233. From Dispanin family. Differentially expressed in psoriasis compared to normal skin |
| *Pygm* | Metabolic pathways, Insulin resistance, Insulin signaling pathway, Starch and sucrose metabolism, Glucagon signaling pathway, Necroptosis | Glycogen phosphorylase, muscle associated. No described association with skin cells or psoriasis |
| *Nnat* | None | Neuronatin. Neuronal developmental and differentiation molecule. Expressed in normal human epidermis and cultured keratinocytes. Overexpressed in psoriasis hyperplasic epidermis. Induced in differentiating keratinocytes. Involved in involucrin expression |
| *Chst3* | Glycosaminoglycan biosynthesis – chondroitin sulfate/dermatan sulfate | Carbohydrate sulfotransferase 3. Synthesizes chondroitin 6-sulfate. Necessary for cartilage formation. *Chst3*-deficient mice showed reduced number of naïve T cells in spleen of. Chondroitin sulfates are expressed in basal keratinocytes. They are found in psoriasis patients but not in healthy donors, in papillary and reticular dermis |
| *Hrc* | None | Histidine rich calcium binding protein. Involved in cardiac, skeletal and arterial smooth muscle development. Expressed in muscle progenitor cells. No described association with psoriasis. |
| *Chad* | PI3K/Akt signaling pathway, Focal adhesion, ECM-receptor interaction | Chondroadherin. Expressed in chondrocytes. Role in bone and cartilage homeostasis. Down-regulated in psoriasis skin |
| *Dmpk* | None | Dystrophia myotonica-protein kinase. Expressed notably in skeletal muscle, skin and cartilage. Expressed by antigen presenting cells. Low expression in keratinocytes. Edits the peptide repertoire presented by major histocompatibility complex class II molecules by professional antigen-presenting cells (APC), favoring presentation of some peptides over others. APC expressing *Dmpk* showed intrinsic kinetic stability of class II–peptide complexes, directly correlated with its levels of expression |
| *Cbs* | Metabolic pathways, Cysteine and methionine metabolism, Glycine, serine and threonine metabolism, Biosynthesis of amino acids | Cystathionine beta-synthase. Vitamin B6-dependent enzyme which converts homocysteine combined with serine into cystathionine. *Cbs*-deficient mice have wrinkled skin with hyperkeratosis and thinning of the dermis. High levels of homocysteine, Cbs ligand, are found in psoriasis patients and might have an impact by inducing ROS, by impairing significantly endothelial function, and by reducing DNA methylation, another factor associated with psoriasis severity. *Cbs*-deficient mice exhibited highly significant induction of the pro-inflammatory cytokines Il-1α, Il-1β and TNFα. Induced in T cells in response to T cell activation. Inhibition limits T cell activation and proliferation |
| *Lmod3* | None | Leiomodin 3 (fetal). Promotes actin nucleation. Important expression in outer cochlear hair cells. Deficiency resulted in a reduction of filamentous actin, elongated cytoskeletal dense bodies. Downmodulated in a murine model of atopic dermatitis |
| *Scn4b* | Adrenergic signaling in cardiomyocytes | Sodium channel, type IV, beta. Involved in cell-cell adhesion in nerve cells. Involved in induced expression of CD69 on CD4^+^ T cells in response to a positive selecting ligand. Expressed in skin and keratinocytes |
| *Mylk2* | Regulation of actin cytoskeleton, Focal adhesion, Platelet activation, Calcium signaling pathway, Apelin signaling pathway, Oxytocin signaling pathway, Glycosaminoglycan biosynthesis – chondroitin sulfate/dermatan sulfate, Gastric acid secretion, cGMP – PKG signaling pathway | Myosin, light polypeptide kinase 2, skeletal muscle. Activated by Ca2^+^. Involved in ERK1/2 and NF-κB pathways. Inhibition in human foreskin fibroblasts caused disassembly of stress fibers and focal adhesions in the central portion of the cell within 1h |
| *Krtap3-3* | None | Keratin associated protein 3-3. Involved in keratinization and developmental biology. Belongs to signature of mouse skin-associated genes. Downmodulated in hair from ILK-deficient mice, which present impaired hair follicle morphogenesis, reduced epidermal adhesion to the basement membrane or compromised epidermal integrity |
| *Jph2* | None | Junctophilin 2. Expressed in skeletal muscle. Essential for proper maintenance of the junctional membrane structure and excitation-contraction coupling. Mutations associated with hypertrophic cardiomyopathy in humans, a disease which could be associated with, or even triggered by, psoriasis. Found differentially expressed in psoriasis skin lesions |
| *Casq1* | None | Calsequestrin 1. Main Ca2^+^-binding protein located in the lumen of sarcoplasmic reticulum terminal cisternae. Down-modulated in skin from mouse atopic dermatitis model compared to healthy controls. Belongs to signature of mouse skin-associated genes |
| *Cgn* | Tight junction | Cingulin. Tight junction actin-binding protein. Expressed in human skin endothelial cells. Overexpression induces tight junction formation and strengthens barrier function. Expressed in epidermis and keratinocytes, and involved in tight junction formation |
| *Stra6* | None | Stimulated by retinoic acid gene 6. Important for the homeostasis of vitamin A and retinoic acid. Constitutively expressed in human epidermal keratinocytes and dermal fibroblasts. Deficiency in HaCaT cell line induces increased proliferation *in vitro* and massive epithelial thickening upon *in vivo* transfer to SCID mice. Deficiency in human organotypic 3D skin models induced a significantly thicker epidermis and enhanced expression of activation, differentiation and proliferation markers, which could be reversed by administration of free retinol. Alterations in retinoid metabolism, signaling and concentrations in psoriasis |
| *Eef1a2* | Legionellosis, RNA transport | Eukaryotic translation elongation factor 1 alpha 2. No expression in keratinocytes. Essential for post-weaning survival in mice. Differentially expressed in psoriasis skin lesions. Expressed in 3D organotypic human skin culture and modulated by IL-31 treatment |
| *Pvalb* | None | Parvalbumin. Mostly cytosolic Ca2^+^-binding protein, serving as a magnesium/calcium buffer. Expressed in developing mouse skin but not in adult skin |
| *Inmt* | Tryptophan metabolism, Selenocompound metabolism | Indolethylamine N-methyltransferase. Degrades histamine in human skin. Expressed by skin fibroblasts |
| *Nr1d1* | Circadian rhythm | Nuclear receptor subfamily 1, group D, member 1. Also known as Rev-erbα. Member of the nuclear receptor superfamily and key circadian clock repressor. Expressed in human hair follicles and keratinocytes. Exerts cell-autonomous inhibitory effects on proliferation and differentiation of myogenic precursor cells which inhibit muscle regeneration in vivo. Inhibits IL-6 and CCL2 gene expression in murine macrophages. Downmodulates IL-17A and IL-17F. Regulate responses of mouse macrophages to complex tissue damage signals and wound repair |
| *Pdk2* | None | Pyruvate dehydrogenase kinase, isoenzyme 2. Most predominant isoforms in skeletal muscle. Role as promoter of classical proinflammatory activation of macrophages. Induced expression in normal oral keratinocytes by cigarette smoke extract. Expressed in 3D skin organotypic constructs |
| *Srgap1* | Axon guidance | SLIT-ROBO Rho GTPase activating protein 1. Important role in neurite outgrowth and axon guidance. Inhibits stromal cell-derived factor-1α-induced chemotaxis of neutrophils during endotoxin-induced lung inflammation. Second most down-regulated gene in psoriasis-like skin lesions in K14-*Angptl6* transgenic mice relative to wild-type littermates. Expressed in 3D skin organotypic constructs |
| *Bco1* | Metabolic pathways, Retinol metabolism | Beta-carotene oxygenase 1. Cleaves beta-carotene into a precursor of vitamin A. Different isoform found in skin. Expressed in many human epidermal cells, including keratinocytes, and in skeletal muscle. Induced expression in skin from UV-treated hairless mice. Downmodulated in skin of an atopic dermatitis mouse model compared to healthy controls. Mutation associated with hypercarotenemia and vitamin A deficiency, and psoriasis skin lesions |
| *Krt32* | None | Keratin 32. Expressed in hair follicles. Regulated by PPARγ, an inhibitor of keratinocyte proliferation which promotes epidermal differentiation. Involved in hair inner root sheath differentiation. Expression in human basal cell carcinoma keratinocytes correlate with enhanced differentiation and limited proliferation. Found downregulated in keratinocyte SRF-deficient mice, which showed disrupted epidermal barrier function leading to early postnatal lethality |
| *Krtap11-1* | None | Keratin associated protein 11-1. Hair follicle specific gene, expressed only in mouse skin, especially in cortical cells of the hair shaft. In cultured keratinocytes, transgenic expression product was strictly regulated by the keratinization process and proteasome-dependent protein elimination. Downregulated in skin of an atopic dermatitis mouse model compared to healthy controls. Expressed in developing mouse skin. |
| *Adh1* | Metabolic pathways, Glycolysis/gluconeogenesis, Drug metabolism – cytochrome P450, Metabolism of xenobiotics by cytochrome P450, Retinol metabolism, Chemical carcinogenesis, Tyrosine metabolism, Fatty acid degradation | Alcohol dehydrogenase 1 (class I). Catalyzes ethanol and retinol oxidation, and plays an important role in retinoic acid production. During late fetal development, transcripts were found essentially in the epidermis. Expressed in the differentiated viable epidermis, fibroblasts, endothelial cells, as well as the upper third of keratinocytes around the hair shaft, and lymphocytes. Downmodulated in psoriasis skin. Polymorphism associated with a risk of developing psoriasis. Downregulated in glucocorticoid receptor-deficient mice, which present impaired epidermal barrier formation, abnormal keratinocyte differentiation, hyperproliferation, and stratum corneum fragility |
| *Asb14* | None | Ankyrin repeat and SOCS box-containing 14. Associated with cell development and structure, cell growth and differentiation, and cell signaling. Unknown function in the skin |
| *Ckm* | Metabolic pathways, Arginine and proline metabolism | Creatine kinase, muscle. Catalyzes the reversible transfer of the gamma phosphate from ATP to creatine forming the high energy compound creatine phosphate. A patient with long lasting psoriasis and multiple sclerosis showed high levels of plasma creatine kinase. Major isoenzyme in normal, uninvolved psoriatic and mouse skin. Increased expression in wounded skin, and in mice and human psoriasis. Both EGF and TGF-α induced a transition from the muscle (*Ckm*) to the brain isoform and histologically induced abnormal differentiation of keratinocytes. Expression localized exclusively within the epidermis and in hair follicles. Help to protect skin from UV damage. Shift from muscle to brain isoform during psoriasis |
